# Evaluation of Oral Health Status and Treatment Needs of Children with Congenital and Acquired Heart Disease

**DOI:** 10.3390/jcm13144060

**Published:** 2024-07-11

**Authors:** Tulin Tasdemir, Gizem Erbas Unverdi, Elif Ballikaya, Ebru Aypar, Hayrettin Hakan Aykan, Tevfik Karagoz, Meryem Uzamıs Tekcicek

**Affiliations:** 1 Department of Pediatric Dentistry, Faculty of Dentistry, Hacettepe University, Ankara 06100, Turkey; erbasgizem@gmail.com (G.E.U.); eyildirim@hacettepe.edu.tr (E.B.); meryemtekcicek@gmail.com (M.U.T.); 2 Pediatric Cardiology Department, Faculty of Medicine, Hacettepe University, Ankara 06100, Turkey; ebruaypar@gmail.com (E.A.); h_h_aykan@yahoo.com (H.H.A.); ktevfik07@gmail.com (T.K.)

**Keywords:** caries, congenital heart disease, oral health status, oral hygiene, teeth

## Abstract

**Objective**: To evaluate the oral health status and treatment needs of children with congenital and acquired heart disease. **Methods**: This descriptive study included 301 children aged 5–14 from June 2022 to June 2023. Heart conditions were classified by congenital/acquired status and severity. The children’s sociodemographic characteristics, medical and dental history, tooth brushing habits, and non-nutritional habits (bruxism, nail-biting, thumb-sucking, etc.) were evaluated. Oral health assessments including caries, oral hygiene, enamel defects, and dental treatment needs-related indices were recorded. **Results**: The mean age was 8.95 ± 2.91 years, and 271 (90%) of the children had congenital heart disease. The children with moderate and severe heart disease had significantly higher decayed/missing/filled surfaces (dmfs) (*p* = 0.038) and pulp exposure (*p* = 0.015) compared to the children with mild heart disease. According to the International Caries Detection and Assessment System II (ICDAS II) index, which included initial caries lesions, there were no caries-free children and 75.7% had extensive caries. The mean plaque index and gingival index were found to be 1.18 ± 0.38 and 0.69 ± 0.53, respectively. Enamel defects were observed in 15.9%. The Treatment Needs Index (TNI) was 85.8% for the primary teeth and 88.9% for the permanent teeth. The Care Index (CI) was 12.4% for the primary teeth and 10.8% for the permanent teeth. **Conclusions**: Children with congenital and acquired heart disease exhibit a high prevalence of untreated dental caries, gingivitis, and plaque accumulation, with a high need for dental treatments. Dentists should prioritize addressing these issues to prevent the risk of infective endocarditis (IE) and improve oral health outcomes in this population.

## 1. Introduction

Heart diseases are caused by structural or functional anomalies in the cardiovascular system, which may be congenital or acquired. The incidence of congenital heart disease (CHD) has been reported to vary between approximately 4 to 50 per 1000 live births [1]. It has been reported that the oral and dental health of children with CHD and acquired heart disease (AHD) is poorer than that of healthy children for various reasons, such as the constant use of sugar-containing drugs; developmental enamel lesions, which are reported to be more common in children with heart disease; and neglect of oral hygiene due to more concern and attention towards the heart disease in the child [2,3,4]. Therefore, early evaluation and therapeutic planning are extremely important.

CHD is the most common risk factor for infective endocarditis (IE) in children [5]. Infection of oral origin has been reported to be associated with 14–20% of IE cases, and gram-positive streptococci are responsible for 50% of these infections. In addition to invasive dental treatments, procedures such as chewing, brushing teeth, and flossing, and foci of oral infection such as periodontal diseases or gingival abscess, have also been claimed to be potential sources of spontaneous bacteremia. The presence of periodontal diseases due to poor oral hygiene can increase the risk of bacteremia through inflamed gingival blood vessels or pocket formation [6,7].

This study aimed to evaluate the oral and dental health status and treatment needs of children with CHD or AHD and also to investigate the potential association between the severity of heart disease and oral/dental health findings. The H1 hypothesis of the present study was established as “There is a difference in the oral and dental health status and treatment needs among children with CHD when grouped by severity of disease”.

## 2. Materials and Methods

This descriptive study was conducted between June 2022 and June 2023 in accordance with the Declaration of Helsinki on medical research ethics and the STROBE guidelines. Informed consent was obtained from the parents of all participants. Hacettepe University Health Sciences Research Ethics Committee (GO 22/421) approval was obtained.

The study population included children aged 5 to 14 years diagnosed with CHD or AHD, who were followed up at the Pediatric Cardiology Department of Hacettepe University Faculty of Medicine and referred to the Department of Pediatric Dentistry in the Faculty of Dentistry of the same university for oral examinations. Participation was on a voluntary basis, and informed consent was obtained. Patients who were uncooperative for an oral examination, those with genetic syndromes, and those without family consent to participate in the study were excluded.

It was primarily planned to investigate the differences and relationships between the independent groups (mild, moderate, and severe, depending on the severity of heart disease) in this study. The sample size was calculated using the G Power 3.1.9.2 Programme (Dusseldorf. University, Kiel, Germany), with the *p*-value, power, and effect size set at 0.05, 95%, and 0.25, respectively. The minimum sample size was calculated to be 252 children.

Data collection involved a survey and examination form prepared by the research teams from both departments. Sociodemographic information about the children and their families, medical and dental history, and brushing habits were collected via face-to-face interviews and recorded by the researcher using a questionnaire. The oral examinations were performed by a single, experienced pediatric dentist (TT) at Hacettepe University, Department of Pediatric Dentistry, and the intra-examiner reliability for caries diagnosis was calculated using the Kappa test. Eighteen children were examined using the International Caries Detection and Assessment System II (ICDAS II) index, with a one-week interval between examinations. In cases where the examiner was uncertain during the oral examinations, the patients were reassessed by two other experienced researchers (G.E.U. and E.B.), each with over ten years of experience, to reach a consensus. To evaluate dental caries, the dmft/DMFT and dmfs/DMFS (decayed/missing/filled teeth and surfaces for primary and permanent dentition, respectively) indices were used to identify cavitated caries, while the ICDAS II index [8] was employed for detecting both initial lesions and cavitated lesions. In this study, the oral effects and severity of untreated dental caries were assessed using the PUFA index (pulpal involvement (P/p), ulceration caused by dislocated tooth fragments (U/u), fistula (F/f), and abscess (A/a)) [9]. The Silness–Löe plaque [10] and Löe–Silness gingival [11] indices were employed to evaluate plaque accumulation and the severity of gingival inflammation on referenced teeth (Ramfjord teeth). The Modified Developmental Enamel Defect Index (mDDE) was used to identify enamel defects in children [12] in order to examine the frequency of this condition among them and its distribution according to the severity of heart disease. For diagnosing and evaluating molar incisor hypomineralization (MIH), the criteria published by Weerheijm et al. [13] in 2003 were utilized. The Treatment Needs Index (TNI) [14] and Care Index (CI) [15] derived from the dmft/DMFT index were used to determine the need for treatment. The TNI and CI were defined using the following formulae:

$$TNI=\frac{decayed\;teeth}{decayed\;teeth+filling\;teeth}\times (100)$$

$$CI=\frac{filled\;teeth}{decayed\;teeth+missing\;teeth+filling\;teeth}\times (100)$$

The ages of the children were modified from the dentition status grouping used in the study by Pollard et al. [16] and divided into three age groups: 5–6 years, 7–10 years, and 11–14 years. Patients’ heart diseases were categorized into two groups: CHD and AHD. The severity of heart disease in the 271 children with congenital heart disease was classified into three categories: mild, moderate, and severe, according to the classification by Warnes et al. [17]. Oxygen saturation values measured by pulse oximeter were used to determine whether patients were acyanotic or cyanotic. In total, 216 patients whose oxygen saturations were recorded in the hospital system were included. Patients with oxygen saturation between 95% and 100% were grouped as acyanotic, and those with oxygen saturation below 95% were grouped as cyanotic [18]. The children participating in the study were divided into subgroups based on factors related to heart disease and oral health. These factors included whether the patient had a history of intensive care unit stay or IE, whether they had a disease other than heart disease, whether they were taking medication that could cause dry mouth, whether the heart disease was congenital or acquired, and whether the patient was acyanotic or cyanotic based on oxygen saturation levels.

### Statistical Analysis

Data analyses were performed using SPSS 21.0. (IBM Corp. Released 2012. IBM SPSS Statistics for Windows, Version 21.0. IBM Corp.: Armonk, NY, USA). Descriptive statistics (percentage, mean, median, standard deviation, minimum and maximum values, and 25th and 75th percentiles) were calculated. A *t*-test was employed for evaluating the relationship between numeric binary variables, and the Mann–Whitney U test was used for non-parametric binary variables. The Kruskal–Wallis test was used for more than two non-parametric variables. For categorical variables, chi-squared test, Fisher’s exact test, and exact test was used to determine if observed differences were statistically significant. The examiner’s internal consistency was determined using Cohen’s kappa analysis. The relationship between the severity of heart disease and various potential factors was evaluated using binary logistic regression analysis. Statistical significance was defined as *p* < 0.05.

## 3. Results

In this study, 301 children who met the inclusion criteria were enrolled, including 30 children with AHD and 271 children with CHD. The data distribution regarding examination findings was evaluated based on the severity of heart disease and the patients’ subgroups. The Kappa value for intra-examiner reliability was found to be 91%. The mean age of the 301 children was 8.95 ± 2.91 years, and 52.5% were male. In total, 62% of the children were diagnosed with heart disease before the age of one. Additionally, 23.3% of the participants had another chronic disease in addition to heart disease. Of the 231 children without comorbidities, 29.4% were using medication for heart disease known to cause dry mouth (Table 1). The impact of intensive care unit stay on dental health was examined in relation to heart disease severity. The patients with severe CHD were significantly more likely to have intensive care unit stay compared to those with mild CHD (Table 2).

Table 3 details the average values of oral health indices (dmft/DMFT, dmfs/DMFS, pufa/PUFA, plaque, and gingival index) by heart disease severity and patient subgroups. A significant difference in dmfs values was observed across the groups, which, upon further analysis via a post hoc test, was attributed to higher dmfs values in the children with severe heart disease compared to those with mild heart disease (*p* = 0.038). Intensive care unit stay was found to be associated with significantly higher dmfs values (*p* = 0.013). The children with no history of IE had significantly higher mean dmfs values than those having a history of IE (*p* = 0.042). The mean dmft and dmfs values of the children with CHD were significantly higher than those with AHD (*p* = 0.038 and *p* = 0.046, respectively). The cyanotic children had significantly higher mean dmfs values compared to the acyanotic children (*p* = 0.050). The children who had a history of IE exhibited significantly higher mean DMFT and DMFS values than those who did not (*p* = 0.016 and *p* = 0.042, respectively). The children with AHD had significantly higher mean DMFT and DMFS values than those with CHD (*p* = 0.016 and *p* = 0.031, respectively). The children with CHD exhibited statistically higher mean plaque index values compared to the children with AHD; similarly, the children without a history of IE had higher mean plaque index values than those with a history of IE (*p* = 0.003 and *p* = 0.001, respectively).

No significant association was found between the severity of heart disease and the increase in the percentage of extensive caries (ICDAS 5–6) according to the ICDAS index (*p* = 0.466). Enamel defects were present in 15.9% of the children and molar incisor hypomineralisation (MIH) in 8.5%. Significantly more enamel defects were observed in the children with severe heart disease compared to those with mild or moderate heart disease (*p* = 0.031). No significant difference was found among the groups when the presence of MIH was evaluated according to the severity of heart disease (*p* = 0.185) (Table 4). The presence of pulp exposure (*p*) was significantly higher in the children with moderate and severe heart disease than in those with mild heart disease (*p* = 0.015). The mean value for pulp exposure in children without a history of IE was significantly higher than in those with a history of IE (*p* = 0.039). Similarly, the mean pufa value was significantly higher in the children taking medicines that cause dry mouth compared to those who were not (*p* = 0.032) (Table 4).

When evaluating the Treatment Needs Index (TNI) and the Care Index (CI) values in relation to heart disease, it was observed that in primary teeth, the children with moderate and severe heart disease had higher TNI and lower CI values compared to those with mild heart disease, but this difference was not significant. Similarly, in permanent teeth, differences between the groups were not significant (Table 5). Analysis of the TNI and CI values according to patient subgroup characteristics indicated that the need for dental treatment was higher and the met restorative dental care was lower in children with CHD compared to those with AHD, as well as in children without a history of IE compared to children with a history of IE. No significant differences were found in the TNI values and CI values for both primary and permanent dentition (Table 5).

Table 6 presents data on the presence of non-nutritional habits and malocclusion by heart disease severity. The children with severe heart disease exhibited significantly more non-nutritional habits than the children with mild heart disease (*p* = 0.050). No significant difference was observed in the presence of malocclusion among the groups (*p* = 0.281).

Binary logistic regression analysis to assess the impact of heart disease severity on various oral health factors revealed that, in the patients with severe heart disease compared to those with mild heart disease, there was an increased likelihood of pulp exposure (OR = 2.54), developmental enamel defects (OR = 2.05), non-nutritional habits (OR = 2.10), and nail-biting habits (OR = 2.12) (Table 7 and Table 8).

## 4. Discussion

Existing research has evaluated the presence of dental caries [19,20,21,22,23,24,25,26], plaque and gingivitis [20,26,27,28], developmental enamel defects [29,30,31], and the clinical consequences of untreated dental caries [32] in children with heart disease. However, there are limited studies evaluating initial caries lesions (ICDAS index), molar hypomineralization (MIH), and treatment needs (TNI) in these children.

The incidence of actively carious deciduous teeth is higher in children with cyanotic heart disease than in children with acyanotic heart disease, according to Berger et al. [28]. The present study confirms this finding, showing higher dmfs values in cyanotic children, most likely hypoxia-induced changes in saliva that lower pH and increase caries risk [33]. Additionally, children with moderate and severe heart disease, who often require medications causing dry mouth, had higher dmfs compared to children with mild heart disease. These findings highlight the complex interplay between heart disease severity, medication side effects, additional disease, and oral health risks.

Studies [16,19,20,21,26,30] comparing caries in healthy children and those with heart disease have reported varying results, and no studies have compared oral health in children according to heart disease status (congenital/acquired). In this study, the children with CHD had significantly higher dmft, dmfs, and plaque index values than the children with AHD. Similarly, dmfs values were found to be significantly higher in the children who stayed in the neonatal intensive care unit compared to the children who did not. Intensive care unit stays, lengthy hospitalizations, and invasive procedures early in life are increased risk factors for caries in children with CHD [34,35,36].

Plaque index (PI) and gingival index (GI) are also indicators of oral health. Although there are studies evaluating the plaque and gingival indices in children with heart disease [20,26,32], research using the Silness–Löe plaque index and Löe–Silness gingival index is limited [27]. In a recent study [27], the mean plaque index for children with CHD was found to be similar to that in the present study, while the mean gingival index in the present study was lower.

PUFA is an index used to assess the presence of oral conditions resulting from untreated caries. In addition to the presence of caries, high pufa index scores increase the risk of developing odontogenic infection [9]. Therefore, the presence of pufa symptoms in the patient increases the risk of developing IE. In the present study, the presence of pulp exposure was significantly higher in the children with moderate and severe heart disease compared to those with mild heart disease. Considering dietary habits as another influencing factor, Schulz-Weidner et al. [37] found that children aged between 2 and 6 years with heart disease consumed more cariogenic food and beverages daily than children in the healthy control group. Despite similar oral hygiene habits among the groups in the current study, the high presence of pulp exposure in the patients with severe heart disease may be attributed to the progression of existing caries, exacerbated by delays in seeking dental treatment by families, rather than to dietary habits or the underlying medical condition.

When evaluating enamel defects in relation to heart disease severity, defects were observed in 14.5% of the children with mild heart disease, 18.8% of those with moderate heart disease, and 25.8% of those with severe heart disease. This suggests that severe heart conditions, which are likely to necessitate intensive care unit stay, could disrupt tooth development more significantly. While Hallett et al. [29] reported a 52% rate of enamel defects in a study conducted in 1992, Sarac et al. [32] reported this rate to be 9% in a study conducted in 2023. This indicates a notable reduction in enamel defects over the years, which possibly reflects advancements in early medical interventions and surgical treatments reducing systemic health impacts like cyanosis [31].

The Treatment Needs Index (TNI) and Care Index (CI) are derived from the DMFT index, which measures current (components of decayed teeth) and past (components of missing and filled teeth) caries experience in permanent teeth. The TNI indicates unmet treatment needs in the population that need to be implemented. It calculates the relationship between untreated decayed teeth and teeth treated with restoration or extraction (due to decay). A decrease in the value of this index towards zero indicates that dental treatment services in the community are good. The CI indicates restorative care that has been performed in a patient, and it is desired to be close to 100% [38]. In the present study, the TNI was found to be 85.8% for primary teeth and 88.9% for permanent teeth. The CI was found to be 12.4% for primary teeth and 10.8% for permanent teeth. While the unmet treatment need (TNI) is quite high in children with heart disease, the met restorative care (CI) is quite low. The CI is indicative of a more radical treatment planning approach for primary teeth in cardiac patients, due to contraindication of pulp treatment in children with heart disease [39]. In this study, the CI value for the permanent dentition was also relatively low. Untreated caries (d) is the predominant factor influencing dmft values, constituting the highest proportion in both primary and permanent teeth [40].

Nail-biting was observed to be 2.12 times higher in the children with severe heart disease than in those with mild heart disease. This may be related to high levels of stress, exacerbated by the increased intensive care required for serious conditions [41]. The increased stress affects not only the children but also their families, with those facing complex CHD bearing more financial and emotional strain [42]. Nail-biting in these children could be a manifestation of this stress. Baydas et al. [43] highlighted a significant relationship between nail-biting habits and an increased prevalence of bacteria such as *Escherichia coli* and *Enterobacteriaceae* in saliva. Furthermore, this behavior can cause trauma to the gingival margin and oral mucosa, providing a favorable environment for bacterial colonization and infection. Consequently, it should be considered that a nail-biting habit may elevate the risk of infection and could also become a risk factor for patients with heart disease [44]. Therefore, addressing the treatment and underlying causes of nail-biting is crucial.

This study provided detailed oral examination findings in a relatively large patient population and addressed the findings by disease severity. Evaluating dental caries in terms of both cavitated and non-cavitated initial lesions, evaluating MIH lesions, showing the outcomes of untreated caries lesions with PUFA indices, and determining the dental treatment needs of these patients were strengths of the study. However, this study should be evaluated within its limitations. The lack of a control group consisting of healthy children, and a lack of assessment dietary habits—an important component of caries risk assessment—were limitations of the study. Additionally, not differentiating between the forms of medication (liquid or solid) in patients using dry mouth medications, and not measuring salivary flow rate for dry mouth, were also limitations of this study. Despite these limitations, the findings of this study will raise awareness among both pediatric cardiologists and dentists. Prospective cohort studies including children with and without CHD may clarify the oral health impacts of heart disease better with a large patient population.

## 5. Conclusions

This paper highlights the importance of oral health in children with heart disease by examining a larger sample with various oral health indices compared to previous studies. Children with heart disease have high rates of untreated tooth decay, which may increase the risk of IE. It was revealed in the present study that children with severe heart disease are more prone to pulp exposure, enamel defects, and non-nutritive habits than those with mild heart disease. Additionally, children with moderate and severe heart disease have a greater need for dental treatment and receive less restorative dental care than those with mild heart disease. Families of these children often face additional health challenges such as recurrent surgeries and intensive care unit admissions, which can lead to oral health being deprioritized. However, preventing complications from poor oral health is crucial, as it significantly impacts overall health and can prevent a vicious cycle. Effective management of these children’s health requires collaboration between pediatric dentists and cardiologists. It is recommended that children with heart disease be referred to a pediatric dentist before age one for a tailored treatment plan.

## Figures and Tables

**Table 1:** Descriptive characteristics of the patients.

Variables	*n*	%
**Age (years)**		
5–6	85	28.2
7–10	111	36.9
11–14	105	34.9
**X ± SD = 8.95 ± 2.91; median = 9.00; 1.−3. quartiles = 6.00–11.00; min–max = 5–14**		
**Gender**		
Male	158	52.5%
Female	143	47.5%
**Heart disease (congenital/acquired)**		
Congenital	271	90.0%
Acquired	30	10.0%
**Age at diagnosis of heart disease**		
Before age one year †	187	62.0%
After age one year	114	38.0%
**Additional disease**		
Yes	70	23.3%
No	231	76.7%
**Using medications associated with dry mouth ††**		
Yes	68	29.4%
No	163	70.6%

X ± SD: mean ± standard deviation. † Includes prenatal, at birth, and 12 months postpartum. †† The evaluation was conducted on a total of 231 patients who had no additional diseases.

**Table 2:** Distribution of intensive care unit stay in children according to the severity of congenital heart disease.

	Severity of Heart Disease	Mild	Moderate	Severe	Total	*p*
Staying in Intensive Care		*n* (%) †	*n* (%) †	*n* (%) †	*n* (%)	
Yes		26 ^a^ (17.1)	12 ^a,b^ (21.1)	20 ^b^ (32.3)	58 (21.4)	0.049 *
No		126 ^a^ (82.9)	45 ^a,b^ (78.9)	42 ^b^ (67.7)	213 (78.6)	

Column percentage. * Pearson’s chi-squared test. † Different superscript letters correspond to statistical difference in the same column.

**Table 3:** Evaluation of some oral health-related indices of the patients according to severity of congenital heart disease and patient subgroup characteristics.

Characteristic	dmft	dmfs	DMFT	DMFS	pufa	PUFA	Plaque Index	Gingival Index
	X ± SD	X ± SD	X ± SD	X ± SD	X ± SD	X ± SD	X ± SD	X ± SD
**Severity of heart disease (*n* = 271)**								
Mild (*n* = 152)	6.32 ± 4.31	14.32 ± 12.36	3.44 ± 3.25	4.66 ± 4.64	0.52 ± 1.14	0.02 ± 0.13	1.18 ± 0.37	0.72 ± 0.54
Moderate (*n* = 57)	7.19 ± 4.95	18.36 ± 17.92	3.33 ± 3.93	5.43 ± 9.18	0.80 ± 1.29	0.10 ± 0.37	1.25 ± 0.33	0.62 ± 0.49
Severe (*n* = 62)	7.67 ± 3.87	18.78 ± 11.58	3.38 ± 3.08	4.63 ± 5.15	0.79 ± 1.04	0.08 ± 0.33	1.21 ± 0.44	0.64 ± 0.53
*p*	0.141 **	0.038 **	0.750 **	0.750 **	0.056 **	0.208 **	0.368 **	0.368 **
**Staying in intensive care unit (*n* = 301)**								
Yes (*n* = 59)	7.70 ± 4.74	19.98 ± 15.53	2.86 ± 2.53	3.98 ± 4.31	0.90 ± 1.60	0.07 ± 0.34	1.23 ± 0.39	0.74 ± 0.55
No (*n* = 242)	6.34 ± 4.26	14.56 ± 12.91	3.71 ± 3.61	5.27 ± 6.49	0.53 ± 0.95	0.05 ± 0.24	1.17 ± 0.39	0.68 ± 0.53
*p*	0.056 *	0.013 *	0.310 *	0.242 *	0.224 *	0.846 *	0.312 ***	0.565 *
**History of IE (*n* = 301)**								
Yes (*n* = 24)	4.54 ± 3.26	9.38 ± 10.35	4.43 ± 2.46	5.90 ± 3.85	0.21 ± 0.43	0.05 ± 0.22	0.91 ± 0.39	0.68 ± 0.57
No (*n* = 277)	6.75 ± 4.42	16.07 ± 13.74	3.47 ± 3.53	4.95 ± 6.35	0.64 ± 1.15	0.05 ± 0.26	1.21 ± 0.38	0.69 ± 0.53
*p*	0.057 *	0.042 *	0.016 *	0.042 *	0.232 *	0.916 *	0.001 *	0.826 *
**Additional disease (*n* = 301)**								
Yes (*n* = 70)	6.30 ± 4.60	16.12 ± 15.09	3.42 ± 3.40	5.46 ± 7.91	0.79 ± 1.44	0.04 ± 0.19	1.20 ± 0.36	0.74 ± 0.49
No (*n* = 231)	6.73 ± 4.33	15.57 ± 13.20	3.60 ± 3.48	4.90 ± 5.50	0.56 ± 1.01	0.06 ± 0.28	1.18 ± 0.40	0.68 ± 0.55
*p*	0.387 *	0.791 *	0.721 *	0.929 *	0.966 *	0.734 *	0.908 *	0.241 *
**Using medications associated with dry mouth (*n* = 231) †**								
Yes (*n* = 68)	7.12 ± 3.91	16.35 ± 12.44	3.45 ± 2.88	4.53 ± 4.65	0.79 ± 1.07	0.02 ± 0.13	1.21 ± 0.39	0.62 ± 0.50
No (*n* = 163)	6.58 ± 4.50	15.26 ± 13.53	3.66 ± 3.73	5.07 ± 5.86	0.47 ± 0.97	0.07 ± 0.32	1.17 ± 0.40	0.70 ± 0.56
*p*	0.442 *	0.306 *	0.837 *	0.788 *	0.032 *	0.243 *	0.414 ***	0.365 *
**Heart disease (congenital/acquired) (*n* = 301)**								
Congenital (*n* = 271)	6.78 ± 4.39	16.11 ± 13.70	3.40 ± 3.34	4.81 ± 5.92	0.64 ± 1.16	0.05 ± 0.26	1.21 ± 0.38	0.68 ± 0.53
Acquired (*n* = 30)	4.84 ± 4.13	11.05 ± 12.45	4.77 ± 4.13	6.88 ± 7.72	0.35 ± 0.67	0.08 ± 0.27	0.97 ± 0.41	0.75 ± 0.53
*p*	0.038 *	0.046 *	0.016 *	0.031 *	0.367 *	0.371 *	0.003 *	0.532 *
**Oxygen saturation level (*n* = 183) ††**								
100–95% (Acyanotic) (*n* = 137)	6.03 ± 4.29	13.73 ± 13.03	3.59 ± 3.50	4.80 ± 5.45	0.56 ± 1.06	0.04 ± 0.23	1.16 ± 0.38	0.67 ± 0.54
≤94% (Cyanotic) (*n* = 46)	7.45 ± 3.33	18.36 ± 8.88	3.41 ± 3.62	4.12 ± 4.55	0.55 ± 1.21	0.00 ± 0.00	1.34 ± 0.45	0.70 ± 0.49
*p*	0.170 *	0.050 *	0.672 *	0.541 *	0.526 *	0.449 *	0.064 ***	0.774 *

X ± SD: mean ± standard deviation. dmft: number of decayed, missing due to caries, and filled teeth in the primary dentition. dmfs: number of decayed, missing due to caries, and filled teeth surface in the primary dentition. DMFT: number of decayed, missing due to caries, and filled teeth in the permanent dentition. DMFS: number of decayed, missing due to caries, and filled teeth surface in the permanent dentition. PUFA/pufa: pulpal involvement (P/p), ulceration caused by dislocated tooth fragments (U/u), fistula (F/f), and abscess (A/a). IE: infective endocarditis. * Mann-Whitney U test. ** Kruskal–Wallis test. *** Sample *t*-test. † The evaluation was conducted on a total of 231 patients who had no additional diseases. †† A total of 183 patients with accessible oxygen saturation values from the hospital registry system were evaluated.

**Table 4:** Distribution of ICDAS II code and the presence of enamel defect, MIH, and pulp exposure by severity of congenital heart disease in the children.

	Congenital Heart Disease Severity								
Characteristic	Mild		Moderate		Severe		Total		*p*
	(*n* = 152)		(*n* = 57)		(*n* = 62)		(*n* = 271)		
	*n* †	%	*n* †	%	*n* †	%	*n*	%	
**ICDAS II code**									
ICDAS 1–2	15 ^a^	9.9	4 ^a^	7.0	2 ^a^	3.2	21	7.7	0.466 *
ICDAS 3–4	24 ^a^	15.8	7 ^a^	12.3	8 ^a^	12.9	39	14.4	
ICDAS 5–6	113 ^a^	74.3	46 ^a^	80.7	52 ^a^	83.9	212	77.9	
**Pulp exposure**									
No	128 ^a^	84.2	41 ^b^	71.9	42 ^b^	67.7	211	77.9	0.015 **
Yes	24 ^a^	15.8	16 ^b^	28.1	20 ^b^	32.3	60	22.1	
**Enamel defect**									
No	130 ^a^	85.5	52 ^a^	91.2	46 ^b^	74.2	228	84.1	0.031 **
Yes	22 ^a^	14.5	5 ^a^	8.8	16 ^b^	25.8	43	15.9	
**MIH**									
No	139 ^a^	91.4	55 ^a^	96.5	54 ^a^	87.1	248	91.5	0.185 **
Yes	13 ^a^	8.6	2 ^a^	3.5	8 ^a^	12.9	23	8.5	

Column percentage. ICDAS II: International Caries Detection and Assessment System II. MIH: molar incisor hypomineralization. * Exact test. ** Pearson’s chi-squared test. † Different superscript letters correspond to statistical difference in the same column.

**Table 5:** Evaluation of Treatment Needs Index (TNI) and Care Index (CI) according to the patients’ subgroups.

	Indices	Primary Dentition				Permanent Dentition			
Patients’ Subgroups		*n*	TNI	*n*	CI	*n*	TNI	*n*	CI
**Severity of heart disease (*n* = 271)**									
Mild (*n* = 152)		111	84.6	113	13.2	89	88.9	89	10.7
Moderate (*n* = 57)		44	90.2	44	8.2	30	94.2	30	5.7
Severe (*n* = 62)		44	90.9	44	8.4	41	89.5	41	10.5
*p* *			0.398		0.445		0.612		0.613
**Staying in intensive care unit (*n* = 301)**									
Yes (*n* = 59)		45	88.4	46	10.1	30	88.6	30	11.3
No (*n* = 242)		172	85.2	173	13.0	152	89.0	152	10.7
*p* **			0.492		0.469		0.915		0.919
**History of IE (*n* = 301)**									
Yes (*n* = 24)		13	68.4	13	31.4	19	77.8	19	22.1
No (*n* = 277)		204	86.9	206	11.2	163	90.2	163	9.5
*p* **			**0.052**		**0.040**		**0.004**		**0.004**
**Additional disease (*n* = 301)**									
Yes (*n* = 70)		53	82.4	53	15.8	44	89.0	44	10.9
No (*n* = 231)		164	86.9	166	11.3	138	88.9	138	10.8
*p* **			0.250		0.227		0.885		0.885
**Using medications associated with dry mouth (*n* = 231) †**									
Yes (*n* = 68)		50	89.8	50	9.7	45	90.9	45	9.0
No (*n* = 163)		114	85.7	116	12.0	93	87.9	93	11.7
*p* **			0.542		0.634		0.336		0.339
**Heart disease (congenital/acquired) (*n* = 301)**									
Congenital (*n* = 271)		199	87.2	201	11.0	160	90.0	160	9.7
Acquired (*n* = 30)		18	70.7	18	27.8	22	80.8	22	19.1
*p* **			**0.035**		**0.031**		**0.020**		**0.020**
**Oxygen saturation level (*n* = 183) ††**									
100–95% (Acyanotic) (*n* = 137)		103	90.9	103	7.8	77	91.1	77	8.5
≤94% (Cyanotic) (*n* = 46)		33	86.3	33	12.1	27	91.9	27	8.0
*p* **			0.466		0.448		0.609		0.605

IE: infective endocarditis. * Kruskal–Wallis test. ** Mann-Whitney U test. † The evaluation was conducted on a total of 231 patients who had no additional diseases. †† A total of 183 patients with accessible oxygen saturation values from the hospital registry system were evaluated.

**Table 6:** Distribution of non-nutritional habits and malocclusion characteristics of the children according to the severity of congenital heart disease.

	Congenital Heart Disease Severity								
Characteristics	Mild		Moderate		Severe		Total		*p* *
	(*n* = 152)		(*n* = 57)		(*n* = 62)		(*n* = 271)		
	*n* †	%	*n* †	%	*n* †	%	*n*	%	
**Non-nutritional habits ††**									
No	103 ^a^	67.8	36 ^a,b^	63.2	31 ^b^	50.0	170	62.7	0.050
Yes	49 ^a^	32.2	21 ^a,b^	36.8	31 ^b^	50.0	101	37.3	
**Bruxism**									
No	135 ^a^	88.8	50 ^a^	87.7	55 ^a^	88.7	240	88.6	0.975
Yes	17 ^a^	11.2	7 ^a^	12.3	7 ^a^	11.3	31	11.4	
**Nail-biting**									
No	119 ^a^	78.3	42 ^a,b^	73.7	39 ^b^	62.9	200	73.8	0.067
Yes	33 ^a^	21.7	15 ^a,b^	26.3	23 ^b^	37.1	71	26.2	
**Malocclusion**									
No	98 ^a^	64.5	33 ^a^	57.9	33 ^a^	53.2	164	60.5	0.281
Yes	54 ^a^	35.5	24 ^a^	42.1	29 ^a^	46.8	107	39.5	

Column percentage. † Different superscript letters correspond to statistical difference in the same column. †† Non-nutritional habits included bruxism, nail-biting, thumb-sucking, pacifier use, bottle use, and lip-biting. * Pearson’s chi-squared test.

**Table 7:** Binary logistic regression analysis of some oral health-related factors of the children according to severity of congenital heart disease.

Oral Health-Related Factors	Odds Ratio	95% (CI)	*p*
**Pulp exposure**			
Mild			**0.017**
Moderate	2.08	1.00–4.29	**0.047**
Severe	2.54	1.27–5.05	**0.008**
**Fistula**			
Mild			0.598
Moderate	1.67	0.57–4.82	0.343
Severe	0.97	0.29–3.24	0.973
**Abscess**			
Mild			0.608
Moderate	1.15	0.28–4.61	0.843
Severe	1.81	0.55–5.96	0.324
**Plaque index**			
Mild			0.867
Moderate	0.99	0.50–1.93	0.983
Severe	1.19	0.60–2.32	0.611
**Gingival index**			
Mild			0.362
Moderate	0.59	0.29–1.21	0.155
Severe	0.91	0.48–1.74	0.793
**Presence of caries**			
Mild			0.280
Moderate	1.45	0.46–4.57	0.525
Severe	3.28	0.72–14.81	0.122
**DMFT**			
Mild			0.687
Moderate	0.75	0.36–1.54	0.444
Severe	0.81	0.42–1.57	0.544
**dmft**			
Mild			0.582
Moderate	1.12	0.54–2.32	0.745
Severe	1.46	0.71–2.98	0.299
**Enamel defect**			
Mild			0.037
Moderate	0.56	0.20–1.58	0.279
Severe	2.05	0.99–4.25	0.052

DMFT: number of decayed, missing due to caries, and filled teeth in the permanent dentition. dmft: number of decayed, missing due to caries, and filled teeth in the primary dentition.

**Table 8:** Binary logistic regression analysis of dental treatment needs and non-nutritional habits of the children by severity of congenital heart disease.

Dental Treatment Needs	Odds Ratio	95% (CI)	*p*
**Endodontic treatment need**			
Mild			0.075
Moderate	2.72	1.08–6.82	0.032
Severe	2.17	0.85–5.54	0.103
**Need for tooth extraction**			
Mild			0.104
Moderate	1.91	1.03–3.55	0.040
Severe	1.43	0.78–2.62	0.247
**Need for orthodontic treatment**			
Mild			0.283
Moderate	1.32	0.70–2.45	0.382
Severe	1.59	0.872.90	0.127
**Need for dental scaling**			
Mild			0.608
Moderate	1.14	0.47–2.77	0.768
Severe	1.51	0.67–3.39	0.319
**Non-nutritional habits** †			
Mild			0.054
Moderate	1.22	0.64–2.31	0.530
Severe	2.10	1.15–3.84	0.016
**Bruxism**			
Mild			0.975
Moderate	1.11	0.43–2.84	0.825
Severe	1.01	0.39–2.57	0.982
**Nail-biting**			
Mild			0.071
Moderate	1.28	0.63–2.60	0.481
Severe	2.12	1.11–4.04	0.022

† Non-nutritional habits included bruxism, nail-biting, thumb-sucking, pacifier use, bottle use, and lip-biting.

## Data Availability

The data supporting the findings of this study are available from the corresponding author upon reasonable request.
